# In Vitro–In Vivo Correlation (IVIVC) Population Modeling for the In Silico Bioequivalence of a Long-Acting Release Formulation of Progesterone

**DOI:** 10.3390/pharmaceutics13020255

**Published:** 2021-02-12

**Authors:** Elena M. Tosca, Maurizio Rocchetti, Elena Pérez, Conchi Nieto, Paolo Bettica, Jaime Moscoso del Prado, Paolo Magni, Giuseppe De Nicolao

**Affiliations:** 1 Department of Electrical, Computer and Biomedical Engineering, University of Pavia, via Ferrata 5, I-27100 Pavia, Italy; paolo.magni@unipv.it; 2 Consultant, I-20100 Milano, Italy; rocchma0@libero.it; 3 ITF Research Pharma SLU, San Rafael 3, 28108 Alcobendas, Spain; eperez@itfsp.com (E.P.); cnieto@itfsp.com (C.N.); jmoscoso@itfsp.com (J.M.d.P.); 4 Italfarmaco S.p.A., Via dei Lavoratori, 54, 20092 Cinisello Balsamo, Italy; p.bettica@italfarmaco.com

**Keywords:** in vitro–in vivo correlation, bioequivalence, vaginal rings, log-acting release, progesterone

## Abstract

Health authorities carefully evaluate any change in the batch manufacturing process of a drug before and after regulatory approval. In the absence of an adequate in vitro–in vivo correlation (Level A IVIVC), an in vivo bioequivalence (BE) study is frequently required, increasing the cost and time of drug development. This study focused on developing a Level A IVIVC for progesterone vaginal rings (PVRs), a dosage form designed for the continuous delivery in vivo. The pharmacokinetics (PK) of four batches of rings charged with 125, 375, 750 and 1500 mg of progesterone and characterized by different in vitro release rates were evaluated in two clinical studies. In vivo serum concentrations and in vitro release profiles were used to develop a population IVIVC progesterone ring (P-ring) model through a direct differential-equation-based method and a nonlinear-mixed-effect approach. The in vivo release, $R_{\mathrm{vivo}}\left(t\right)$, was predicted from the in vitro profile through a nonlinear relationship. $R_{\mathrm{vivo}}\left(t\right)$ was used as the input of a compartmental PK model describing the in vivo serum concentration dynamics of progesterone. The proposed IVIVC P-ring model was able to correctly predict the in vivo concentration–time profiles of progesterone starting from the in vitro PVR release profiles. Its internal and external predictability was carefully evaluated considering the FDA acceptance criteria for IVIVC assessment of extended-release oral drugs. Obtained results justified the use of the in vitro release testing in lieu of clinical studies for the BE assessment of any new PVRs batches. Finally, the possible use of the developed population IVIVC model as a simulator of virtual BE trials was explored through a case study.

## 1. Introduction

Changes in the formulation composition, as well as in the batch manufacturing process, equipment, and site, are commonplace during various stages of drug development or after its regulatory approval. If one or more of these changes occur, in vivo bioequivalence (BE) studies in humans are required, increasing the development costs and time to market. An established in vitro–in vivo correlation (IVIVC) could be used to support biowaivers, thereby largely reducing the regulatory burden, leading to time and cost savings [1,2].

IVIVC has been defined by the U.S. Food and Drug Administration (FDA) as a predictive mathematical model describing the relationship between an in vitro property of a dosage form (e.g., drug dissolution or release profile) and its in vivo response (e.g., concentration–time profile) [3]. IVIVC can be categorized into five different levels: Levels A, B, C, D, and multiple C. The Level A IVIVC, defined as a point-to-point relationship between the in vitro and in vivo profiles of at least three formulations or batches characterized by different release rates, is considered the most informative and is required to obtain a biowaiver. Indeed, through the successful development and application of an adequate Level A IVIVC, the in vivo plasma concentration of a drug can be predicted from the in vitro release data that can be used as a surrogate for the in vivo bioequivalence assessment. 

In 1997, the FDA published a regulatory guidance related to the development, evaluation, and applications of IVIVC for extended-release (ER) oral dosage forms [3]. This guideline, in agreement with most of the older literature, presented the so-called deconvolution- and convolution-based methods as the standard IVIVC technique [4,5], although the development of alternative approaches was encouraged. Since then, the establishment and application of IVIVC have increased and various alternatives to the conventional IVIVC approaches have been introduced [2,6,7,8]. In few but extremely relevant examples, the IVIVC was developed using nonlinear mixed-effect models [9] or physiologically based pharmacokinetic (PBPK) models [10,11,12], in the latter cases frequently implemented in closed software such as GastroPlus^TM^. However, from a recent screenshot of the IVIVC approaches applied to the ER oral drug, it clearly emerged that, even with their promising potential and advantages, the use of these mathematical methods still requires consolidation and harmonization [7].

If no established consensus about approaches to implement an IVIVC for ER oral drugs has been reached, methodologies for ER nonoral dosage forms (e.g., parenteral microspheres and implants, ophthalmic compound, intra-muscular suspensions, as well as transdermal and vaginal products) are still at the very early stages of development [2,13]. To date, there is no regulatory IVIVC guidance for ER nonoral products, and only few study reports are present in the literature. In these examples, the same principles to develop an IVIVC for ER oral products [3] have been applied to dosage forms and the route of administration other than oral [14,15,16].

The vagina is a promising site of systemic and local drug delivery and represents an interesting administration route for compounds with poor oral bioavailability [17,18,19,20]. Between the different types of vaginal drug delivery systems currently available on the market [21,22,23,24], intravaginal rings (IVRs) offer the possibility of a continuous and controlled drug release over an extended time period of several weeks. IVRs are generally used for the steroid administration in hormone replacement therapy or birth control, but more recently, they have witnessed a surge of new interest in the context of the HIV-prevention strategies [25]. Even with the increasing number of marketed products and the numerous IVR formulations that are currently in development, no regulatory guidance on the release tests or IVIVC assessment for vaginal rings are available in the United States, Europe, or Japan. This lack of established guidance and standard procedures makes the development of IVIVC for IVRs, already complex due to the unique anatomy and physiology of the vagina, even more challenging.

In this context, the aim of the present work was to establish a Level A IVIVC between the in vitro release of progesterone vaginal rings (PVRs) and the corresponding serum concentration profiles observed during clinical studies. The proposed IVIVC model was defined through a direct differential equation-based model that, together with the adoption of a nonlinear mixed-effect approach, allowed us to handle the complex dynamics and the variability affecting the in vivo vaginal absorption. The internal and external predictability of the model was carefully evaluated following the recommendations for the Level A IVIVC assessment reported in the regulatory guidance for ER oral drugs [3]. The established IVIVC model resulted in the ability to correctly predict the in vivo concentration–time profiles of PVRs from their respective in vitro release profiles, allowing the use of the in vitro testing as a surrogate for bioequivalence studies testing any new PVR batches (scale-up or post-approval changes). Finally, the possible use of the developed population IVIVC model as a simulator of virtual bioequivalence trials was investigated through a case study. To this aim, estimates of the model-expected in vivo relative bioavailability of two tested PVR batches were reported. 

## 2. Materials and Methods

### 2.1. Experimental Methods 

#### 2.1.1. Drug Product

ITFE-2068 is an intravaginal ring manufactured by ITF Research Pharma S.L.U and is intended for luteal support in Assisted Reproduction Techniques. The ring is a matrix-type reservoir, composed exclusively of silicone and natural progesterone (P), and is designed for the continuous delivery of progesterone for 14 days. All the rings have identical dimensions (58 and 47 mm of external and internal diameter, respectively) and total mass (4.4 g).

#### 2.1.2. In Vitro Release Data

For the in vitro progesterone release determination, individual rings were suspended in glass bottles containing 250 mL of isopropanol/water (60/40). The bottles were introduced in an orbital incubator shaker at 37 °C and agitated at 120 r.p.m. The bottles were sampled at least every 24 h for 15 days with medium reposition. Progesterone was measured with a validated UPLC method using an Acquity HSS C18 1.8 µm: 2.1 × 50 mm column and water/acetonitrile (30/70) as a mobile phase (volume of injection 0.6 µL, flow 0.4 mL/min; column temperature: 30 °C). 

Time courses from 24 up to 360 h of the accumulated amount of progesterone released in vitro were available at different dose levels and batches. For rings charged with 125 and 375 mg of progesterone, additional early sampling timepoints (e.g., 1, 2, 4, 6, 7, 8 h) were considered. Ring batches A–D were characterized by a different release rate depending on the charged dose of progesterone and varying in the range of 44.3–273.1 mg/day^1/2^ (where the release rates were calculated by Higuchi’s formula for release from matrix [26]). A summary of the in vitro information is reported in Table 1.

#### 2.1.3. In Vivo Pharmacokinetic and Release Data

Individual serum profiles of progesterone were collected from 46 subjects enrolled in two clinical studies, ITFE-2068-C1 (n = 30) and ITFE-2068-C1b (n = 16). These were phase I–II single-center, randomized, active-controlled, parallel-group, dose-response clinical trials with the objectives of evaluating the pharmacokinetic (PK), pharmacodynamic, and tolerability profile of PVRs in healthy premenopausal female volunteers. Moreover, they aimed to find the minimum efficacious dose able to maintain an adequate pregestational endometrial transformation during 14 days. Therefore, based on the in vitro release data, three doses (1500, 750, and 375 mg) able to release progesterone for at least 14 days were selected: the 1500 mg dose level was close to the highest progesterone amount compatible with an adequate silicone polymerization; the other two doses (750 and 375 mg) represented 1/2 and 1/4 of the highest dose. To clearly identify the lowest effective dose, a fourth dose (125 mg), expected to be nonefficacious, was also tested in the study ITFE-2068-C1b. This dose was selected based on the in vitro release profile, which showed that it could sustain progesterone release only for one week, after which progesterone was exhausted.

In both the studies, participants were treated with leuprorelin acetate (Ginecrin Depot 3.75 mg, AbbVie Spain), a GnRH agonist, to suppress all endogenous progesterone and estradiol production. Endometrial proliferation was induced with B-estradiol patches (Estradot 75 µg/d, Novartis Farmaceutica) and, after that, ITFE-2068 progesterone rings from different batches and charged with different dose levels were inserted to induce endometrial transformation. 

An information summary of the treatment groups composing the clinical studies is reported in Table 2. 

For the PK analysis, serum progesterone was measured at 0.5, 1, 2, 3, 4, 6, 8, 12, 18, 24, 36, 48, 72, 96, 120, 144, 168, 216, 264, 312, 380, and 408 h post-dosing. Serum progesterone levels were measured using an IMMUNLITE 2000 automated system (Siemens Medical solutions Diagnostics), based on a solid-phase-competitive chemiluminescent immunoassay assay. The assay was previously validated following current bioanalytical method validation guidelines. GnRH agonist treatment produced an almost complete suppression of endogenous production. Indeed, before ring insertion, the median baseline progesterone serum concentration was 0.387 ng/mL (range 0–0.84 ng/mL) to compare with expected values of 5–50 ng/mL in women during mid-cycle. All values were corrected by subtracting the respective baseline values.

After 18+/−1 days, rings were removed. For each subject, the amount of progesterone still present in the ring after removal from the vagina was measured. The total amount of progesterone released within the observation period was assessed by subtracting this quantity from the charged dose.

The studies were conducted following the international recommendations for clinical research gathered in the declaration of Helsinki and its update, the ICH Harmonized tripartite Guidelines for Good Clinical Practice (CPMP/ICH/135/95), guidelines of the Spanish Ministry of Health (RD 223/2004), as well as the Directive 2001/20/EC. Human investigations were performed after approval by the Spanish Drug Agency and the Ethical Committee for Clinical Research of the ‘Hospital Universitario La Paz’ in Madrid.

### 2.2. IVIVC Progesterone Ring Model Building 

A direct, differential-equation-based method was applied to establish the IVIVC [9]. This approach allowed us to directly relate the time profiles of the in vitro releases with the in vivo serum concentration profiles by using a multi-compartment PK model and the corresponding system of differential equations. 

The development of the IVIVC progesterone ring (IVIVC P-ring) model was done in three steps. First, a model for the in vitro progesterone release from the ring, $R_{vitro}\left(t\right),$ was defined based only on the in vitro release tests performed for batches A–D at the different dose levels. Second, a model predicting the in vivo release, $R_{vivo}\left(t\right),$ from the in vitro profile ($R_{vitro}\left(t\right))$ was developed through the introduction of an inhibitory function accounting for the limited solubility of progesterone in the finite volume of vaginal fluids. Finally, a population PK model describing the entire in vivo serum concentration profiles of progesterone was built using a compartmental PK model whose input was the predicted in vivo release $R_{vivo}\left(t\right)$.

#### 2.2.1. In Vitro Release Model 

The in vitro progesterone profiles were described by an immediate release (burst phase) followed by two exponential release phases (a faster and a slower one). The time-profile of the in vitro cumulative progesterone release ($P_{vitro}\left(t\right)$) was represented by the relationship reported in Equation (1):

(1) $$P_{vitro}\left(t\right)=P_{0}+A\left(1-e^{-\alpha t}\right)+B\left(1-e^{-\beta t}\right)$$

where the model parameters were the amount of progesterone immediately released, $P_{0}$ [mg]; the fractions of the fast, A [mg], and of the slow in vitro release, $B=DOSE-P_{0}-A$ [mg]; and the fast and the slow in vitro rates, *α* and *β* [1/h], respectively. 

The in vitro release rate, $R_{vitro}\left(t\right)$, was the first derivative of the cumulative progesterone release, $P_{vitro}\left(t\right)$:

(2) $$R_{vitro}\left(t\right)=P_{0}\delta \left(t\right)+A\alpha e^{-\alpha t}+B\beta e^{-\beta t},$$

where δ(t) is the delta Dirac function.

For each dose level, the in vitro model parameters ($P_{0}$, A, *α*, and *β*) were estimated and the value of B derived. To this aim, Equation (1) modeling $P_{vitro}\left(t\right)$ was identified against the corresponding experimental data. As the individual in vitro profiles overlapped almost perfectly, resulting in a very low inter-ring variability (the average %CV is less than 4% for each dose), for each dose, a unique curve was estimated using a pooled data approach. Model identification was performed in NONMEM using the first-order conditional estimation method (FOCE) algorithm and assuming an additive residual error model (y=f+sd·ε, where *y* is the measurement, f the model prediction, sd a coefficient representing the standard deviation, and ε a standardized random variable normally distributed).

#### 2.2.2. In Vivo Release Model 

**Figure 1:**
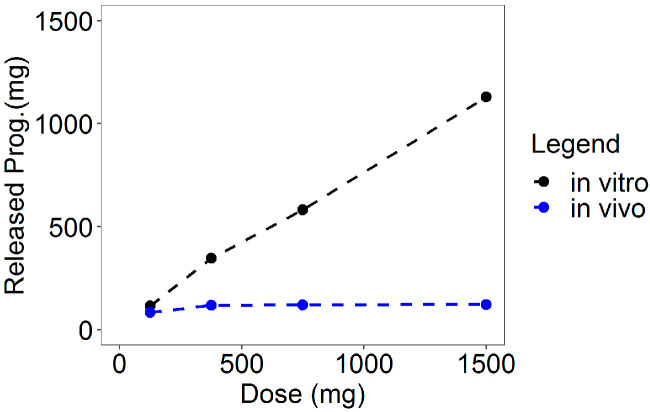
Comparison between the average total amounts of in vitro and in vivo-released progesterone.

To define the in vivo release model for the PVRs, the average total amount of released progesterone at the end of the in vitro release tests and after ring removal at the end of the in vivo clinical trials were compared (see Table 3 and Figure 1). 

From Table 3 and Figure 1, it was evident that, although the observation times of the in vivo studies were slightly longer (three days more) compared to the in vitro experiments, the amounts of in vivo-released progesterone were definitely smaller than the in vitro ones. These differences highlighted an inhibition of the in vivo release due to the limited solubility of progesterone (approximately 12 ng/mL in water; 4 ng/mL in simulated vaginal fluid) in the finite volume of vaginal fluids (0.5–0.75 mL) [20], suggesting that the saturation of the vaginal fluids was the rate-controlling mechanism of the in vivo progesterone release from the rings.

To account for this inhibition, a functional dependence (f), connecting the in vivo release rate with the in vitro profile ($R_{vitro}\left(t\right)$, Equation (2)), was introduced.

(3) $$R_{vivo}\left(t\right)=f\left(R_{vitro}\left(t\right)\right).$$

In particular, an inhibitory function acting on the rate terms of the in vitro release model was used to account for the saturated release observed in vivo in comparison to the in vitro situation. The time-profile of the in vivo release rate of progesterone ($R_{vivo}\left(t\right)$) was, thus, represented by the relationship reported in Equation (4):

(4) $$R_{vivo}\left(t\right)=f\left(R_{vitro}\left(t\right)\right)=P_{0}\delta \left(t\right)+A\alpha_{vivo}e^{-\alpha_{vivo}t}+B\beta_{vivo}e^{-\beta_{vivo}t}$$

where $\alpha_{vivo}=\alpha \left(1/\left(1+\gamma \right)\right)$ and $\beta_{vivo}=\beta \left(1/\left(1+\gamma \right)\right)$; the parameters $P_{0}$ [mg], A [mg], B [mg], *α* [1/h], and *β* [1/h] were the same as those of the in vitro release model; and the nonnegative parameter γ is the in vitro–in vivo inhibitory factor. 

Integrating the in vivo release rate of progesterone, $R_{vivo}\left(t\right)$, the cumulative release, $P_{vivo}\left(t\right)$, was derived (Equation (5)):

(5) $$P_{vivo}\left(t\right)=P_{0}+A\left(1-e^{-\alpha_{vivo}t)}\right)+B\left(1-e^{-\beta_{vivo}t}\right)$$

To identify the value of the in vitro–in vivo inhibitory factor γ, the model prediction of the in vivo cumulative release of progesterone at 408 h ($P_{vivo}\left(408h\right)$) was matched to the average amount measured after the ring removal at the end of the clinical trial ($P_{vivo,observed}$):

(6) $$P_{vivo}\left(408h\right)=P_{0}+A\left(1-e^{-\alpha 408h\left(1/\left(1+\gamma \right)\right)}\right)+B\left(1-e^{-\beta 408h(1/\left(1+\gamma \right)}\right)=P_{vivo,observed}\;$$

For each dose level, a different estimate of γ was obtained by solving Equation (6) through the *uniroot*() function implemented in R. In order to describe the dependence of γ on the dose, a second-order polynomial model was used:

(7) $$\gamma =\;\gamma \left(DOSE\right)=c_{0}+c_{1}DOSE+c_{2}DOSE^{2}$$

where DOSE is the amount of progesterone charged in the ring expressed in mg, and the coefficients *c*_0_, *c*_1_ and *c*_2_ were estimated using an ordinary least squares approach against the value previously obtained on the single dose levels.

#### 2.2.3. Pharmacokinetic Model

The time profiles of the progesterone serum concentration were described by a compartment PK model receiving as input the in vivo release rate, $R_{vivo}\left(t\right)$. 

A two-compartment model with two parallel absorptions, one for the amount of progesterone immediately released and one for the delayed biexponential release phase, and a linear elimination was adopted. 

**Figure 2:**
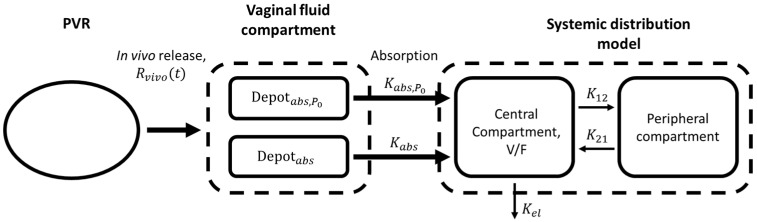
Schematic representation of the in vitro–in vivo correlation (IVIVC) P-ring model. From the ring, the progesterone is released in the vagina lumen according to the in vivo release rate $R_{vivo}\left(t\right).$ The immediately released amount $P_{0}$ (${\mathrm{Depot}}_{abs,P_{0}}\;compartment)$ is absorbed with a constant rate $K_{abs,P_{0}}$; instead, the remaining released progesterone $\left({\mathrm{Depot}}_{abs}\;compartment\right)$ is absorbed with a slower rate $K_{abs}$. After absorption, the system distribution of progesterone is described by a linear two-compartment model.

A schematic representation of the whole IVIVC P-ring model is reported in Figure 2. The parameters composing the IVIVC P-ring model are summarized in Table 4 together with the data needed for the identification. 

A population approach was developed to describe the individual amounts of released progesterone and the corresponding serum concentration profiles. In particular, the inter-individual variability was modeled assuming that the individual parameters were log-normally distributed, e.g., *P_i_* = *θ* exp(*η_i_*), where *θ* is the typical population value and *η* a normally distributed random effect with zero mean and variance ω(*P*). In particular, to account for the inter-individual variability affecting the in vivo progesterone release, a log-normally distributed random effect was introduced also on the in vitro–in vivo inhibitory factor γ:

(8) $$\gamma_{i}=\left(c_{0}+c_{1}DOSE+c_{2}DOSE^{2}\right)\cdot exp\left(\eta_{\gamma ,i}\right),$$

where the coefficients $c_{0},c_{1}$ and $c_{2}$ were fixed to the previous estimates. A typical value (*θ)* and variance (ω) of model parameters were estimated simultaneously against individual serum concentrations and amounts of released progesterone from the two clinical studies (no inter-study variability was included) at the four dose levels (125, 375, 750 and 1500 mg). During the model identification, for each dose level, parameters $P_{0}$, A, B, α and β were kept fixed to the values obtained on the corresponding in vitro data. 

Separate individual error models were used for the serum concentration and the amount of the in vivo released progesterone; in both the cases, an additive residual error model was used. Identification of the population model was performed in NONMEM using the SAEM algorithm.

### 2.3. Evaluation of the IVIVC P-Ring Predictability

Once the IVIVC P-ring model had been developed and completely identified, its ability in predicting the in vivo performance of the PVRs from their in vitro release data was evaluated, adopting the principles for the Level A IVIVC assessment reported for ER oral drugs [3]. Based on the FDA regulatory guidance, depending on the intended application of the IVIVC and on the therapeutic index of the drug products, the internal and external predictability of the IVIVC model must be evaluated considering an adequate range of in vitro release rates and manufacturing changes. In accordance with the more restricting FDA recommendations, the consistency of the IVIVC was tested with the ring batches A–D that showed different in vitro release rates (Table 1). Moreover, both the internal and external predictability of the IVIVC P-ring model was evaluated.

#### 2.3.1. Internal Predictability

Assessment of the internal predictability relates to evaluating how the model is appropriate to describe the data used to define the IVIVC. The approach recommended by FDA guidance is to predict the entire serum concentration profile from the in vitro release data and, then, to compute the percentage Prediction Error (PE) on the area under the curve (*AUC*) and the maximum concentration level (*C_max_*):

(9) $$\;\%PE_{\theta }=\frac{\theta_{obs}-\theta_{pred}}{\theta_{obs}}\times 100,\;\mathrm{where}\;\theta =AUC\;or\;C_{max}$$

The acceptance criteria by the FDA are an average absolute PE of 10% or less for *AUC* and *C_max_* with an individual PE for each batch/release rate lower than 15%. 

According to FDA guidelines, diagnostic plots evaluating the model predictions of the serum concentration profiles of progesterone and the absolute PEs on *AUC* and *C_max_*, average and for each batch/dose level, were used to establish the internal predictability of the IVIVC P-ring model.

#### 2.3.2. External Predictability

The assessment of the external predictability relates to how well the IVIVC model predicts additional data that have not been involved in developing the correlation. Similarly to the internal predictability, the acceptance criteria recommended by FDA are based on the evaluation of the absolute PEs on the *AUC* and *C_max_* that are required to not exceed 10%.

To this scope, the final IVIVC P-ring model was trained only on a subset of the whole dataset, and data relative to batch *B* at dose 375 mg were set aside as an external test set. First, the IVIVC P-ring model was re-estimated on data from batches A, C and D at 125, 750 or 1500 mg doses, and its internal predictability was re-assessed also on the reduced training dataset. Then, the obtained parameter estimates were used to predict serum concentration profiles (500 individual replicates) at 375 mg from the corresponding in vitro release profile. External Visual Predictive Check (VPC) and absolute %PEs on the average *AUC* and *C_max_* were used to assess the external predictability of the model. 

#### 2.3.3. In Vitro Release Profile Comparison

If a Level A IVIVC is achieved, in vitro release profiles can be confidently used as a surrogate for the in vivo bioequivalence in place of clinical BE studies for certain pre- and post-approval changes (e.g., manufacturing site or process changes) [3]. 

To this aim, difference (*f*_1_) and similarity (*f*_2_) factors that originate from a simple model-independent approach can be used to compare in vitro release profiles of two batches (reference and test) [27]. The difference factor (Equation (10)) calculates the percent difference between the two curves at each time point and is a measurement of the relative error between the two curves:

(10) $$f_{1}=\left\{\left[\sum_{t=1}^{n}\left|R_{t}-T_{t}\right|\right]/\left[\sum_{t=1}^{n}\left|R_{t}\right|\right]\times 100\right\}$$

where *n* is the number of time points, $R_{t}$ is the release value of the reference batch at time t, and $T_{t}$ is that of the test batch. The similarity factor (Equation (11)) is a logarithmic reciprocal square root transformation of the sum squared error and is a measurement of the similarity in the percent dissolution between the two curves:

(11) $$f_{2}=50\times log\left\{{\left[1+\left(1/n\right)\sum_{t=1}^{n}{\left(R_{t}-T_{t}\right)}^{2}\right]}^{-0.5}\times 100\right\}$$

Based on FDA acceptance criteria, $f_{1}$ values up to 15 (0–15) and $f_{2}$ values greater than 50 (50–100) ensure sameness or equivalence of the two in vitro release profiles.

## 3. Results

### 3.1. Identification of the IVIVC Progesterone Ring Model

#### 3.1.1. In Vitro Release Model 

For each dose level, the in vitro release model was successfully identified against the corresponding in vitro cumulative release profiles of progesterone. The obtained release curves were able to describe accurately all the profiles observed at the different doses with a CV of parameter estimates always below 5% (see Table 5). At the highest doses, 750 and 1500 mg, the lack of observations in the first 24 h hampered the estimation of the immediate release parameter $P_{0}$, which was therefore kept fixed at the value 9.45 mg, estimated for the 375 mg dose.

**Figure 3:**
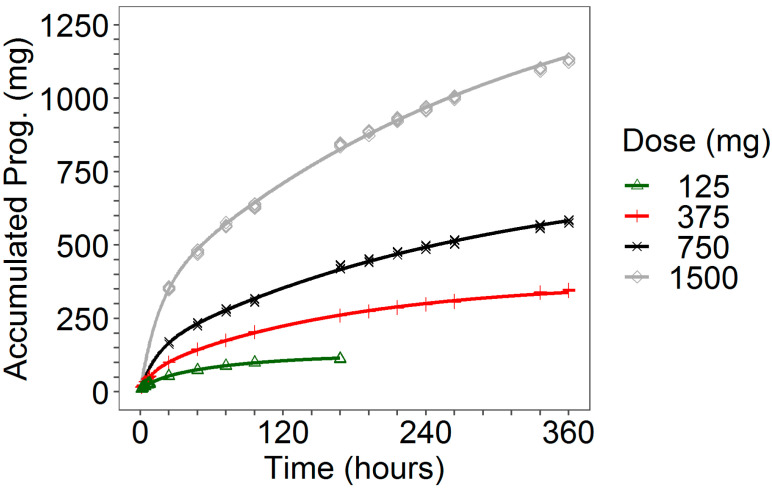
Observed in vitro individual data and fitted cumulative release profiles for all dose levels.

**Figure 4:**
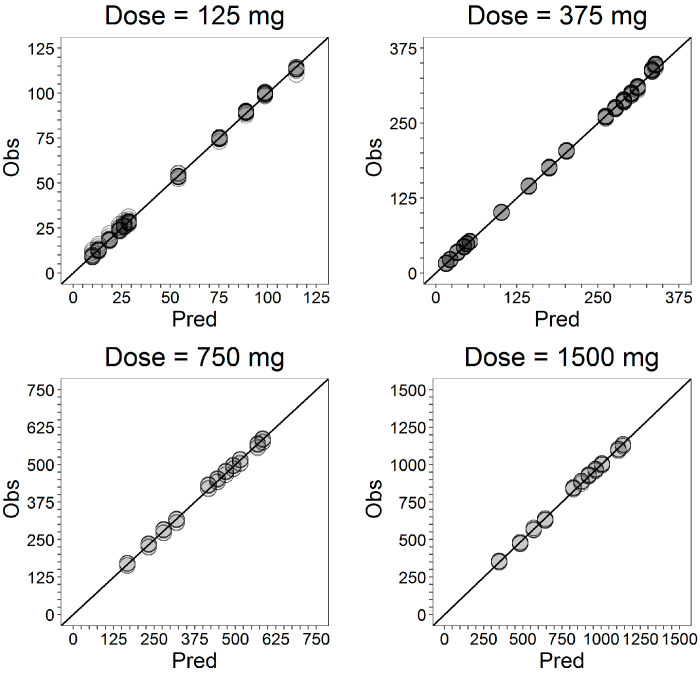
Goodness-of-fit plots of the in vitro release model stratified on the dose level. For each dose level, model predictions are plotted against individual experimental data.

The fitted cumulative release curve is plotted in Figure 3 together with individual experimental data. For each dose level, the goodness-of-fit plots are reported in Figure 4. 

#### 3.1.2. In Vivo Release Model

The introduction of the inhibitory function on the exponential rate terms of the in vitro release model allowed the relationship between the in vitro and the in vivo progesterone release profile to be captured well. For each dose level, the value of the in vitro–in vivo inhibitory factor γ was identified by solving Equation (6). Results are reported in Table 6.

**Figure 5:**
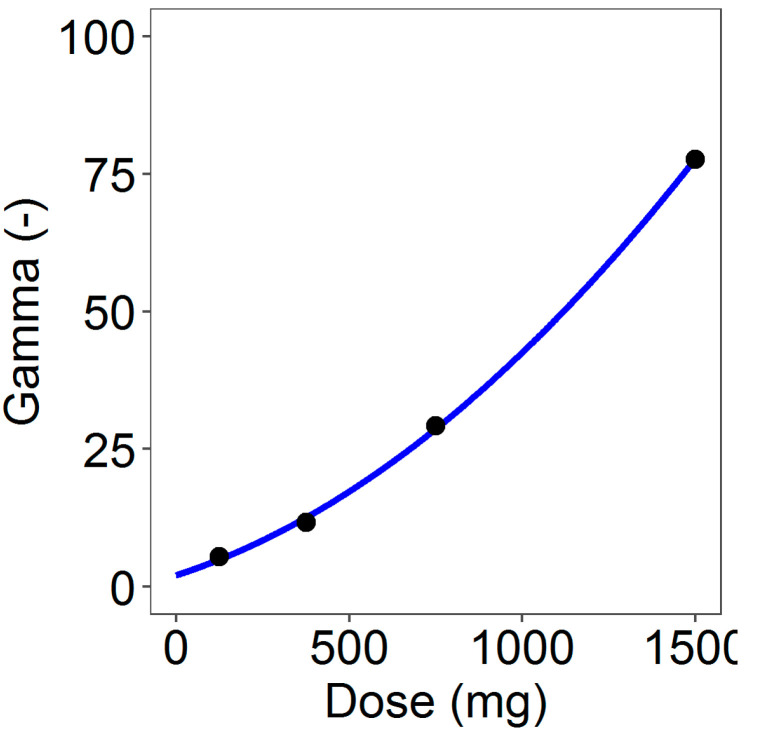
Fitted γ=γ(DOSE) curve describing the dependence of the in vitro–in vivo inhibitory factor on the dose.

Obtained estimates of γ (Table 6) were used to identify the coefficients *c*_0_, *c*_1_ and *c*_2_ of the second-order polynomial (Equation (7)) describing the dependence of the parameter γ on the dose level. Figure 5 shows the fitted γ curve defined by the parameters (SE between brackets) *c*_0_ = 2.116 (1.58), *c*_1_ = 0.020 (5.180 × 10^−5^), and *c*_2_ = 2 × 10^−5^ (3.010 × 10^−6^).

#### 3.1.3. Population IVIVC Progesterone Ring Model

The population IVIVC P-ring model was successfully identified against data relative to the amounts of released progesterone and the corresponding serum concentration profiles at the four dose levels. Random effects were first considered for the γ factor and for all the PK model parameters (data not shown). However, variance ω(K_abs_) was estimated with a very low precision and the individual estimates of K_abs_ showed a very small inter-individual variability (CV% < 3%). Therefore, it was decided to consider the alternative statistical model in which all the individuals share the same K_abs._

The estimates of the population parameters are reported in Table 7. All the parameters were estimated with good precision. 

**Figure 6:**
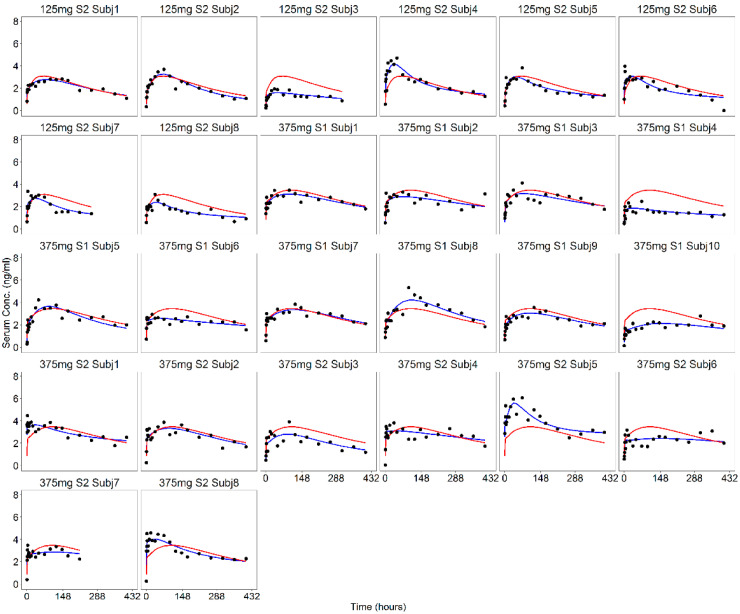
Model predictions of the individual (blue lines) and population (red lines) serum concentration profiles at 125 and 375 mg dose levels together with observations (black dots).

**Figure 7:**
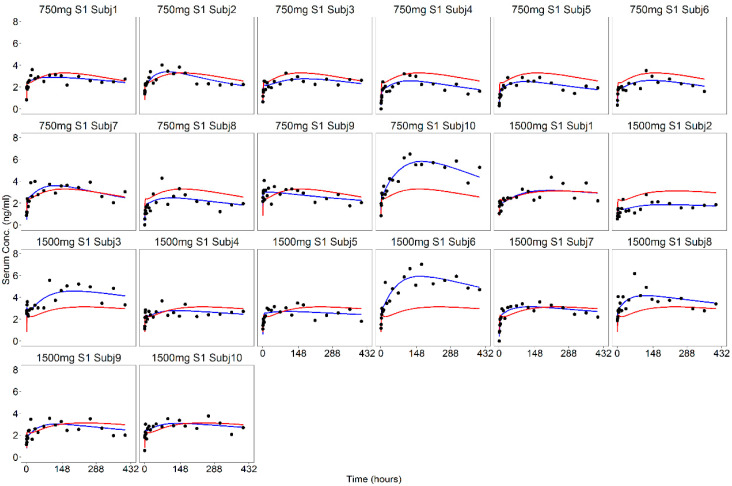
Model predictions of the individual (blue lines) and population (red lines) serum concentration profiles at 750 and 1500 mg dose levels together with observations (black dots).

In Figure 6 and Figure 7, the individual fits of the progesterone serum concentration profiles are plotted at all the dose levels. The good agreement between the observed and predicted profiles showed that the proposed model was able to adequately describe the serum concentration of progesterone from the in vitro release data. Only a slight underestimation of the higher serum concentrations observed during the first hours occurred. 

**Figure 8:**
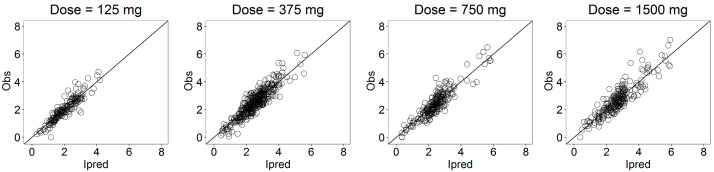
Goodness-of-fit plots for the serum concentration profile of progesterone stratified on the dose level.

**Figure 9:**
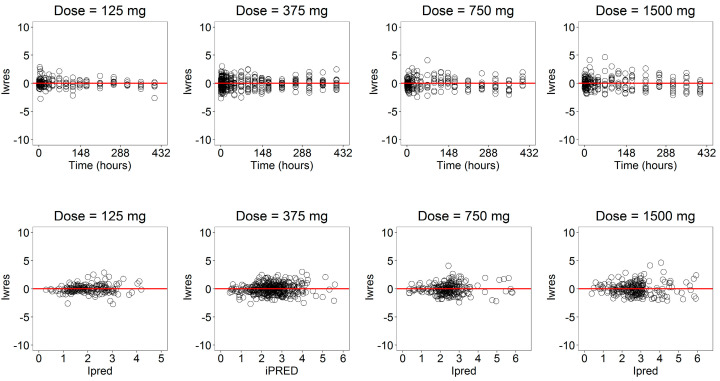
Weighted residuals for the serum concentration of progesterone are plotted against time (**upper** panels) and individual prediction (**lower** panels) stratified on the dose levels.

**Figure 10:**
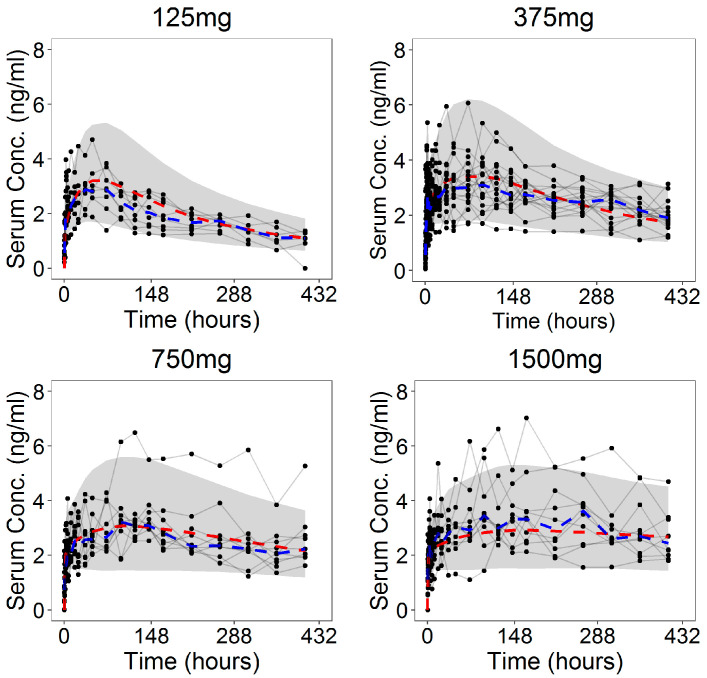
Visual predictive check (VPC) plots (500 individuals for dose level) for the progesterone serum concentration profiles stratified on the dose level: The 90% confidence interval (grey area) is shown together with the observed data (black dots); dashed lines represent the theoretical (red) and experimental (blue) median.

The goodness-of-fit (GOF) plots (Figure 8) suggested that the model was unbiased and the residual error plots (Figure 9) showed a symmetric distribution around zero and no systematic residual trends. Additionally, VPC plots (500 simulations for each dose level) are reported in Figure 10 stratified on dose levels, demonstrating that both typical and inter-individual dynamics of progesterone serum concentration were captured well by the IVIVC P-ring model.

**Figure 11:**
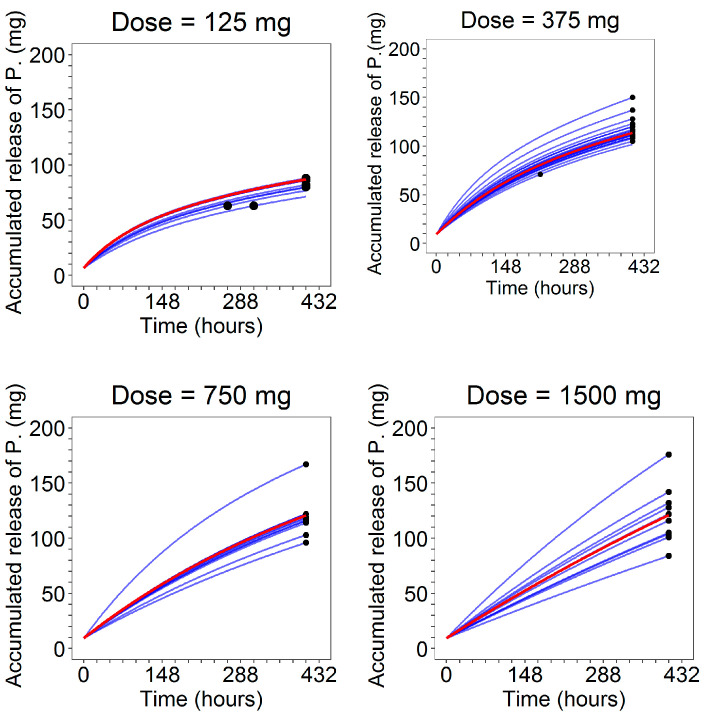
Model predictions of the individual (blue lines) and population (red lines) cumulative release of progesterone together with the observed total amount released at the end of the studies (black dots) stratified on dose level.

Finally, in Figure 11, the fit plots of the amount of cumulative release of progesterone, in which the model predictions were superimposed on the observed total amount released at the end of the studies, are reported.

### 3.2. Evaluation of the IVIVC Progesterone ring Model Predictability

#### 3.2.1. Internal Predictability

As recommended in the FDA guideline [3], the internal predictability of the IVIVC P-ring model was first assessed. The population IVIVC P-ring model was used to derive the individual serum concentration profiles of progesterone from the corresponding in vitro release, and diagnostic plots (Figure 6, Figure 7 and Figure 8) were used to evaluate its performances. As already discussed, for each dose level, the model predictions were in good agreement with observations showing that the proposed model was able to adequately predict the progesterone serum concentration from the in vitro release data.

Then, the prediction errors on *AUC* and *C_max_* were computed, and are reported in Table 8 and Table 9, respectively. For both the PK metrics, the average absolute PE and individual PEs for each dose level were derived; in particular, for *AUC*, which was considered the most informative parameter, various sampling times were taken into account. 

**Figure 12:**
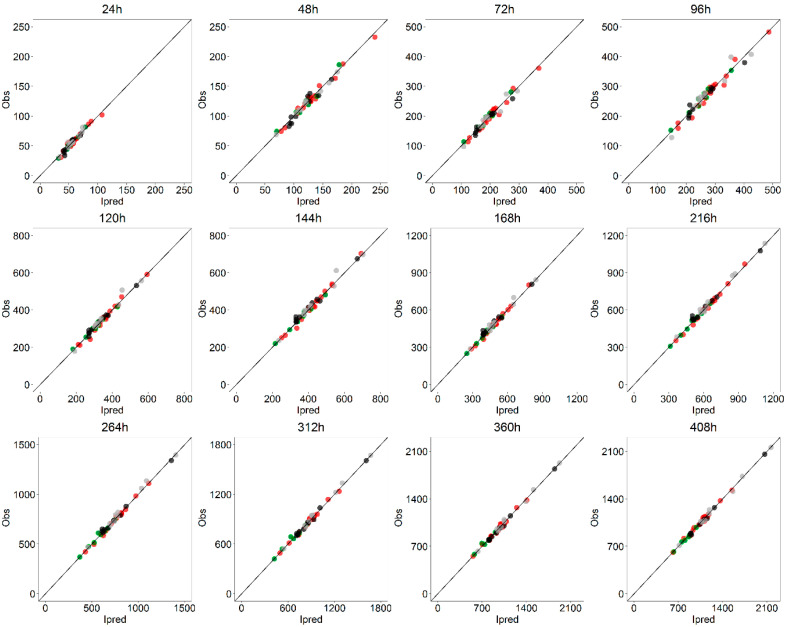
Goodness-of-fit plots for AUC(0-t): Individual observed and model-predicted AUC(0,t) at various sampling times are displayed stratified on dose level by color (green = 125, red = 375, black = 750, grey = 1500 mg).

For each dose level, the absolute PE on *AUC* (0–408 h) is less than 2% (Table 8). Furthermore, the PEs on AUC(0-t) at various sampling times is always less than 7% with an average value always under 5%. The excellent model performances in correctly predicting progesterone exposures are further confirmed by the GOF plots for *AUC* (Figure 12) in which individual AUC(0-t) at various sampling times are shown.

The average PE on *C_max_* slightly exceeds the threshold of 10%. In this case, the higher errors are due to the variability in the data. Indeed, the observed T_max_ of the in vivo serum concentration was affected by a coefficient of variation higher than 75%. 

#### 3.2.2. External Predictability

The external predictability of the IVIVC P-ring model was assessed using data relative to batch B at 375 mg dose as an external test set and the remaining data as the training dataset on which the model parameters were re-estimated. 

To completely identify the model on the internal dataset, first, coefficients *c*_0_, *c*_1_ and *c*_2_ of the second-order polynomial describing the dependence of the γ factor on the dose level were re-estimated using γ values only at the 125, 750 and 1500 mg dose level (see Table 6). Obtained estimates were, then, kept fixed during the identification of the population model against data relative to the amount of released progesterone and the corresponding serum concentration profiles at the three considered dose levels. The parameter estimates are reported in Table A1 in Appendix A. A graphical evaluation of model performance together with obtained values for the absolute PEs on *AUC* and *C_max_* confirmed the internal predictability of the IVIVC P-ring model also on the reduced dataset (see Appendix A). 

**Figure 13:**
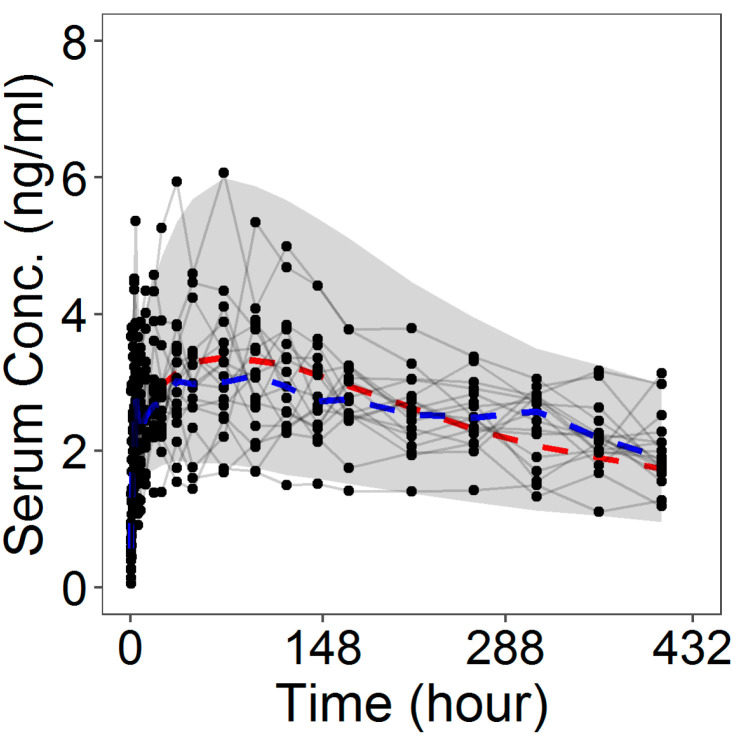
External VPC plot at 375 mg dose level. Simulations of progesterone serum concentrations at dose level 375 mg performed by the IVIVC P-ring model identified on data relative to doses 125, 750, and 1500 mg. The 90% confidence interval (grey area) is shown together with the observed data (black dots); dashed lines represent the theoretical (red) and experimental (blue) median.

Once assessed that the model was adequately identified on the training dataset, the obtained parameters estimates were used to predict the entire serum concentration profiles of progesterone at 375 mg from the corresponding in vitro release profile. Notably, 500 individual replicates were simulated and compared to the observed serum concentrations of progesterone through an external VPC plot (Figure 13). 

The absolute PE on the average AUC(0-t) is reported in Table 10 for the latest four sampling times (264, 312, 360 and 408 h). It is always less than 10% and less than 3% at the end of the experiment. Furthermore, the absolute PE on the average *C_max_* was equal to 5.52. 

Results obtained on the external dataset at 375 mg confirmed the previous finding and demonstrated that the established IVIVC met the predefined internal and external validation criteria.

## 4. Discussion

Methodologies for the development and evaluation of IVIVC models are an active area of investigation and a variety of approaches with different degrees of complexity are possible and potentially acceptable. IVIVC principles have been mostly applied to oral products and, in 1997, a specific guidance for ER oral drugs was been established by regulatory authorities [3]. However, due to the continuous pharmaceutical advancements on drug formulation and administration device, there exists a need to develop methodologies and standards for the development of an IVIVC model of nonoral delivery systems, for which no regulatory guidelines and only sparse literature examples are currently available [13]. 

The objective of this work was the development of a mathematical model able to establish an adequate Level A IVIVC between the in vitro release of PVRs and the corresponding serum concentration profiles in humans. A direct, differential-equation-based method and a nonlinear-mixed-effect approach were adopted to develop the IVIVC P-ring model following the same principles generally used to define an IVIVC for ER oral dosage forms [3].

More in detail, in this study, different in vitro release rates obtained at 125, 375, 750 and 1500 mg dose levels were considered. At each dose level, the observed release profiles from the progesterone rings exhibited bi-exponential decays that overlapped almost perfectly. Hence, within each dose level, the individual in vitro release time courses were pooled together and modeled without taking into account inter-ring variability. An initial burst phase was observed in the release profile of the two lower dose levels (125 and 375 mg) for which early sampling timepoints were available. This immediate faster release is due to the progesterone at or near the surface of the matrix ring that has a relatively small diffusional pathway to overcome in order to be released [28]. The parameter P_0_ of the in vitro model was introduced to provide an estimate of this quantity. However, at the higher dose levels (750 and 1500 mg), the lack of observations in the first 24 h hampered the estimation of P_0_ that was then fixed to the 375 mg dose value without significantly affecting the quality of the GOFs. 

**Figure A5:**
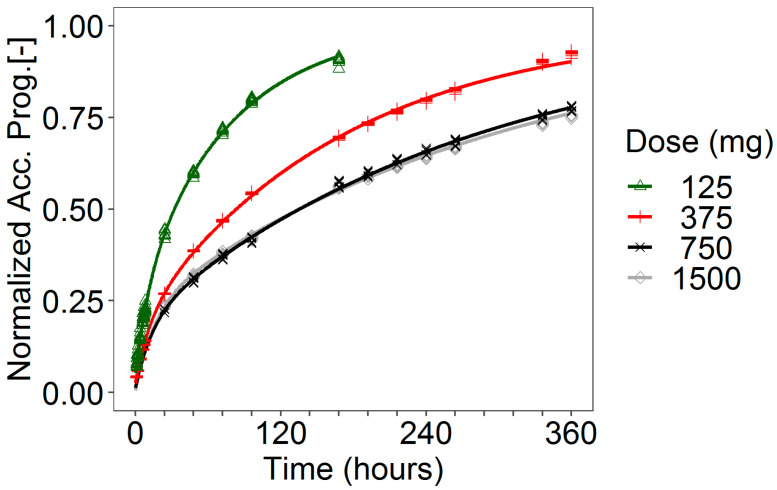
Observed in vitro individual data and fitted cumulative release profiles normalized by the progesterone dose loaded in the ring for all dose levels.

A lack of dose proportionality was observed in the in vitro progesterone release, as it is evident from the release rates computed by Higughi’s formula (see Table 1) and from the dose-normalized in vitro profiles (Figure A5 of Appendix A). This is likely due to the progesterone diffusion process across the ring. Mathematical models accounting for the diffusion step, as well as for the other mechanistic processes affecting the progesterone delivery from the ring, are available in the literature [29,30,31,32]. However, because of the scope of this work, the modeling efforts were focused on obtaining a simple progesterone release function, $R_{vitro}\left(t\right),$ to subsequently use as input rate for the PK model. Despite its empirical structure, the selected bi-exponential model (Equation (1)) was able to capture almost perfectly the in vitro release data (Table 5, Figure 3 and Figure 4). 

A comparison of the in vitro and the in vivo cumulative release at the end of the experiments gave evidence of a decreased in vivo release rate compared to that in vitro (see Table 3). In addition, the total amount of the in vivo released progesterone within the observation period resulted similarly for the three higher dose rings irrespective of the amount charged in the ring. This was caused by the inhibition of the progesterone release due to the vaginal fluid saturation. Indeed, in vivo, the progesterone has to be released from the ring into the vaginal fluids from which it has to be absorbed through the vaginal epithelium. As the daily production of vaginal fluids is approximately 6 mL/day with a constant volume of 0.5–075 mL and the progesterone solubility is very low [20,33], the system is saturated and progesterone solubility results in the rate-controlling mechanism. Drug saturation of vaginal fluids was reached and maintained already with the 375 mg rings, and then no further significant release and absorption was reached by increasing the progesterone in the rings. This inhibition was modeled in the in vivo release model by a suitable inhibition function driven by a dose-dependent in vitro–in vivo factor γ acting on the exponential release rates of the in vitro model. Thus, the in vivo release in input to the PK model was obtained by combining the in vitro release model (for any specific dose) with the inhibition function governed by the parameter γ. 

The PK model incorporated two absorption mechanisms, one ($K_{abs,P_{0}}$) for the immediate progesterone release and the other ($K_{abs}$) for the remaining part of the release. A two-compartment model was assumed for progesterone kinetics in serum. Unlike from the in vitro data, the serum progesterone levels showed different profiles among the subjects with a significant intra- and inter-individual variability. These findings were in agreement with the PK variability reported for the drug administered through the vaginal route and represented an intrinsic characteristic and limitation of this type of administration [28]. Indeed, several physiological factors, such as changes in the epithelial thickness, alteration of the volume and pH of the vaginal fluids, can potentially affect progesterone release from the ring and alter its absorption rate [20,28,34]. A population (nonlinear-mixed effect) approach was used to account for this relevant in vivo variability. The simultaneous fitting of the in vivo parameters yielded very satisfactory results at all doses in terms of both residuals and visual predictive checks. 

Even if progesterone is characterized by a nonnarrow therapeutic index, in accordance with the more restricting FDA recommendations [3], both the internal and external predictability of the IVIVC P-ring model were evaluated. First, the internal validity of the model was confirmed by GOF comparing the predicted and observed curves and the very low PE on AUCs. At any sampling time, the average (across all subjects and doses) PE on AUC(0-t) was less than 5% (Table 8). At the latest times (t ≥ 144 h), the individual PE for each dose was less than 3%. A higher error was observed for *C_max_* whose PE on average was 16.4% (Table 9). This was somehow expected in view of the marked variability in the individual serum profiles, with observed T_max_ values in the range of 3–408 h. Such a *C_max_* PE below 20% was taken as satisfactory even if the FDA guideline for ER oral drugs suggests an average PE lower than 10% with 15% as a maximum bound for individual PEs. In fact, it has to be considered that this kind of formulation is somehow atypical with respect to guidelines for oral administration and that progesterone is a nonnarrow therapeutic index drug. To further confirm the predictive capability of the model, an external validation was set-up. The data relative to batch *B* at dose 375 mg were left apart as a test dataset, and the population IVIVC P-ring model was re-estimated using only data at doses 125, 750 and 1500 mg (ring batches A, C and D). Then, using the in vitro model derived from the 375 mg data, the obtained population model was used to simulate the serum profile of 500 distinct individuals. Validation was performed by means of external VPCs and the PE on the average AUC(0-t) and *C_max_*. In particular, all the subjects fell within the 90% predicted intervals of the VPCs, and the PE on AUCs and *C_max_* were all below 10%. Based on the previous results and observations, the proposed IVIVC P-ring model was considered meeting all the FDA validation criteria and can be used to support in vivo bioequivalence studies. 

Adequately predicting the in vivo performances from in vitro release data for vaginal drug delivery systems, such us PVRs, is extremely challenging due to a multitude of factors including the vaginal physiology, the complex dynamics of vaginal absorption, the significant inter-individual variability of the PK, the absence of compendial apparatus and methods for the in vitro release testing [28], as well as the inability of directly measuring the in vivo release in the vagina during the whole study period. For this reason, as in the majority of IVIVC applications, the progesterone serum concentration–time profiles were used as surrogates to reflect the in vivo drug release. Here, in addition to the serum concentration levels, the total amounts of released progesterone at the end of the study were available, providing a fundamental piece of information for the complete definition of the proposed IVIVC P-ring model. First, similar amounts of progesterone remaining in the rings in the in vivo experiments at the three higher doses (375, 750 and 1500 mg) suggested the consideration that the absence of dose-proportionality observed in the corresponding serum progesterone exposures could be essentially due to factors inhibiting the in vivo release (like solubility in the vagina). Secondly, they allowed the successful identification of the in vitro–in vivo inhibition release model. Finally, although for practical reasons, the data on the in vivo-released amount of progesterone were available only at the end of the trials, the goodness of the fittings suggested the exclusion of the presence of important transient nonlinear behaviors.

Another issue that further increased the challenging IVIVC development was the in vitro release system adopted for PVRs that was only partially physiological. Indeed, in aqueous systems (or vaginal fluids), the in vitro progesterone release from rings loaded with 375, 750 and 1500 mg was identical and corresponds to the saturation of the receiving medium. Therefore, for quality control, it was needed to include surfactants that increased the progesterone solubility in the receiving medium and allowed us to discriminate releases from rings charged with different progesterone amounts. The establishment of an IVIVC based on a nonfully physiological in vitro release system represents an important achievement of this modeling exercise.

Finally, a case study, exemplifying the possible use of the developed IVIVC P-ring model to the BE assessment of any new batch of progesterone rings, is proposed here. A new batch of vaginal rings (batch E) charged with 375 mg of progesterone was available. Batches B and E were expected to be bioequivalent and were considered as the reference and test batch, respectively. As a Level A IVIV relationship was defined by the IVIVC P-ring, clinical BE studies can be avoided and the BE assessment can be based only on the comparison of the in vitro release profiles. Thus, the time course of the accumulated amount of progesterone released in vitro was also derived for 12 rings of the test batch (batch E). Comparison of the in vitro release profiles of the two batches at the 375 mg dose level was performed by computing the difference and similarity factors (Equations (10) and (11)). Obtained values for both the parameters ($f_{1}=3.84$ and $f_{2}=99.96$) fell within the FDA criteria allowing the assessment of the BE of the batches. 

In addition to the standard model-independent procedure, the population IVIVC P-ring model approach developed here allows us to perform a virtual BE trial providing estimates of the relative bioavailability in vivo (F = AUC_test_/AUC_ref_). With this scope, the in vitro release model (Equation (1)) was identified on the in vitro release data of the test batch, and parameters were estimated (Appendix B). Then, a 500 individual population treated with vaginal rings from the reference batch (batch B at 375 mg) was simulated using the IVIVC P-ring model and the correspondent release profile from batch B. A second independent population (500 subject) treated with vaginal rings of the test batch (batch E) was simulated using the same IVIVC P-ring model with the exception of the in vitro release parameters identified on batch E. Generally, in vivo BE is assessed in a unique population receiving treatments from reference and test batches in two different moments. However, because no information about intra-individual variability was available from the clinical data, a virtual BE trial was performed under the conservative assumption that intra-individual variability was equal to the inter-individual variability (even if it was expected to be lower). The simulated concentration profiles for the reference and test populations were then used to derive the correspondent *AUC*. Finally, the relative bioavailability in vivo (F = AUC_test_/AUC_ref_ = 0.913) and its 90% confidence interval (90%CI = [0.878, 0.948]) were computed. From the virtual BE trial, it emerged that the reference and test batches respected the 80–125% bioequivalence criteria, further confirming the bioequivalence of this case study. 

## 5. Conclusions

In summary, a Level A IVIVC model for PVRs was developed based on a direct differential-based equation method and a population nonlinear mixed-effect approach. The internal and external predictability of the proposed IVIVC P-ring model was evaluated and its good performances as a tool for the assessment of the in vivo bioequivalence from in vitro release studies were demonstrated through the application on a case study. 

Thus, this work represents an interesting example of a validated IVIVC exploitable as a surrogate of bioequivalence studies, for a complex nonoral (vaginal) ER dosage form for which no clear regulatory guidelines and less experience are available. The proposed approach, based on an ad-hoc developed population model, is a suitable alternative to the standard convolution- and deconvolution-based IVIVC methods, as well to the PBPK-based approach generally developed on closed software applications. 

## Figures and Tables

**Table 1:** Analyzed in vitro release experiments.

Dose Level [mg]	Batch	N. of Replicates	Calculated Release Rate [mg/day^1/2^]
125	A	12	44.3
375	B	12	91.7
750	C	6	145.4
1500	D	6	273.1

**Table 2:** Information summary of the considered clinical studies.

Clinical Trial	Dose [mg]	Batch	N. of Subjects
ITFE-2068-C1b	125	A	8
ITFE-2068-C1b	375	B	8
ITFE-2068-C1	375	B	10
ITFE-2068-C1	750	C	10
ITFE-2068-C1	1500	D	10

**Table 3:** Comparison between the total amount of released progesterone in vitro and in vivo.

Dose [mg]	In Vitro (over 15 Days)		In Vivo (over 18 Days)	
	Average Released Progesterone [mg]	Percentage of Total Released Progesterone [%]	Average Released Progesterone [mg]	Percentage of Total Released Progesterone [%]
125	114	91.20	84.40	67.52
375	346.40	92.37	117.82	31.42
750	582.04	77.61	119.30	15.91
1500	1129.62	75.31	121.00	8.07

**Table 4:** Structural parameters of the IVIVC P-ring model.

Parameter	Unit	Description	Data Used for the Identification
$P_{0}$	mg	Immediate in vitro release	In vitro release profile
A	mg	Fraction of fast in vitro release	In vitro release profile
B	mg	Fraction of slow in vitro release	In vitro release profile
α	1/h	Fast in vitro release rate	In vitro release profile
β	1/h	Slow in vitro release rate	In vitro release profile
γ	-	in vitro–in vivo inhibitory factor, defined as $c_{0}+c_{1}DOSE+c_{2}DOSE^{2}$	Average amounts of the in vivo cumulative release (end of the clinical study)
$c_{0},c_{1},c_{2}$	-	Polynomial coefficient of the γ function	γ estimates for single dose levels
$K_{abs,P_{0}}$	1/h	Absorption rate for $P_{0}$	In vivo serum concentration profile and cumulative release
$K_{abs}$	1/h	Absorption rate for the bi-exponential phase release	In vivo serum concentration profile and cumulative release
$K_{el}$	1/h	Elimination rate constant from the central compartment	In vivo serum concentration profile and cumulative release
*V*/F	L	Apparent volume of distribution	In vivo serum concentration profile and cumulative release
$K_{12}$	1/h	Transfer rate constant from central to peripheral compartment	In vivo serum concentration profile and cumulative release
$K_{21}$	1/h	Transfer rate constant from peripheral to central compartment	In vivo serum concentration profile and cumulative release

**Table 5:** Parameter estimates of the in vitro release model.

Parameters	125 mg	375 mg	750 mg	1500 mg
	Estimate [CV%]	Estimate [CV%]	Estimate [CV%]	Estimate [CV%]
$P_{0}$ [mg]	6.62 [5]	9.5 [2]	9.5 [fix]	9.5 [fix]
A [mg]	26.1 [3]	59.1 [1]	130 [1]	318 [1]
B [mg]	92.28 [-]	306.45 [-]	610.55 [-]	1172.55 [-]
α[1/h]	0.0843 [4]	0.0846 [1]	0.0721 [1]	0.0656 [3]
β [1/h]	0.013 [1]	0.0059 [<1]	0.00361 [1]	0.0033 [<1]
Error (sd)	1.13 [16]	2.84 [2]	6.55 [10]	10.5

**Table 6:** Estimates of the γ parameter.

Dose [mg]	Estimate
125	5.437
375	11.643
750	29.219
1500	77.601

**Table 7:** Parameter estimates of the population IVIVC P-ring model.

Population Parameters		
Parameter [Unit]	Typical Value (CV%)	Inter-Individual Variance (CV%)
γ [-]	$c_{0}+c_{1}DOSE+c_{2}DOSE^{2}$	0.0579 (12)
$K_{abs,P_{0}}$ [1/h]	0.7180 (16)	0.2770 (17)
$K_{abs}$ [1/h]	0.1990 (24)	*-*
$K_{el}$ [1/h]	0.0241 (5)	0.0092(64)
*V*/F [L]	3450 (9)	0.0596 (22)
$K_{12}$ [1/h]	0.0259 (19)	0.1570 (45)
$K_{21}$ [1/h]	0.0264 (59)	3.8600 (28)
**Residual Variability (Variance)**		
$var_{conc}$	0.2040 (2)	
$var_{amount}$	0.0002 (71)	

**Table 8:** Absolute prediction errors (PEs) on AUC[0-t].

		Absolute PE [%]											
	Time [h]	24	48	72	96	120	144	168	216	264	312	360	408
Dose [mg]													
**125**		4.53	4.08	3.95	3.72	2.56	1.82	1.51	1.51	1.87	2.19	1.89	1.46
**375**		4.54	3.28	4.56	4.39	3.14	2.19	2.02	1.96	1.80	1.61	2.10	1.85
**750**		6.12	5.58	3.66	3.57	3.06	3.52	3.68	2.48	1.93	1.78	1.77	1.59
**1500**		3.98	1.88	5.04	5.67	3.79	2.49	2.77	2.68	2.38	1.79	1.74	1.53
**Average**		4.79	3.71	4.30	4.34	3.14	2.51	2.50	2.16	1.99	1.85	1.87	1.61

**Table 9:** Absolute %PEs on *C_max_*.

Dose [mg]	Absolute %PE
**125**	15.57
**375**	14.56
**750**	18.16
**1500**	17.36
**Average**	16.41

**Table 10:** Absolute PEs on average AUC[0-t] at 375 mg dose level.

	Absolute PE			
**Time [h]**	**264**	**312**	**360**	**408**
**PE [%]**	7.36	3.75	3.75	2.95

**Figure A1:**
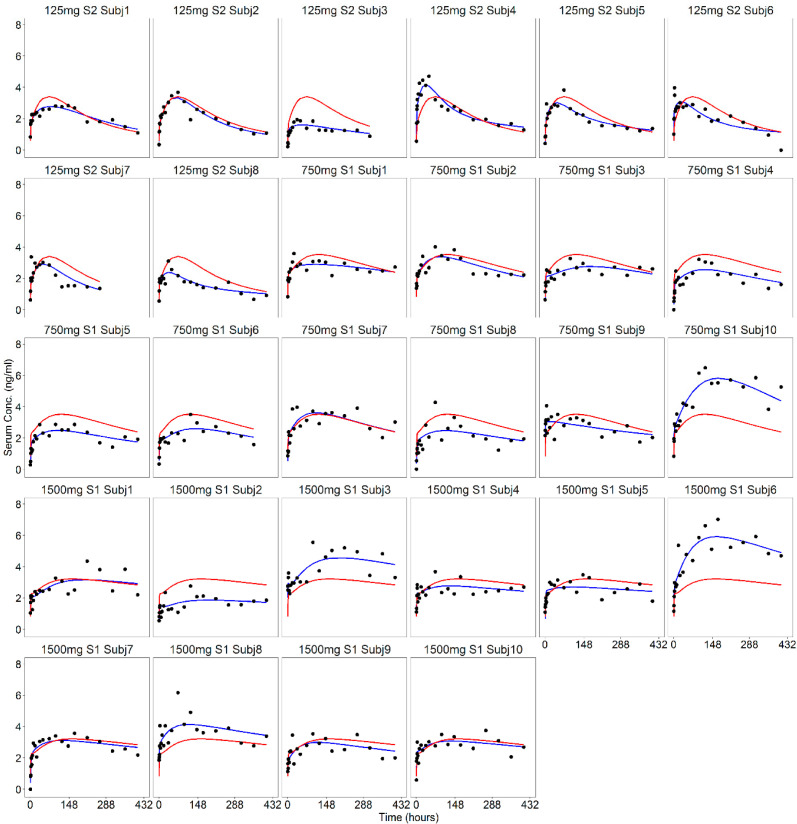
Model predictions of the individual (blue lines) and population (red lines) serum concentration profiles at 750 and 1500 mg dose levels together with observations (black dots).

**Figure A2:**
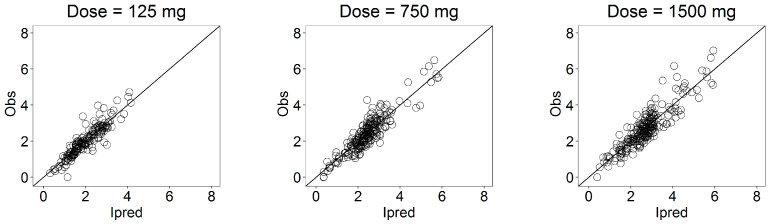
Goodness-of-fit plots for the serum concentration profile of progesterone stratified on the dose level.

**Figure A3:**
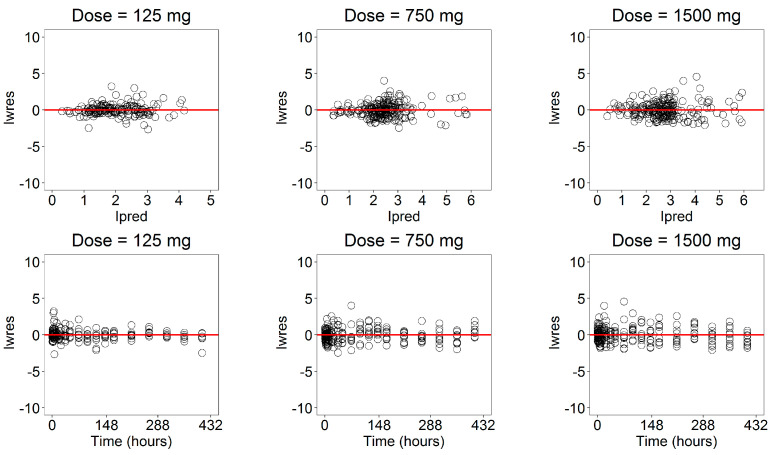
Weighted residuals for the serum concentration of progesterone plotted against time (**upper panels**) and individual prediction (**lower panels**) stratified on the dose levels.

**Figure A4:**
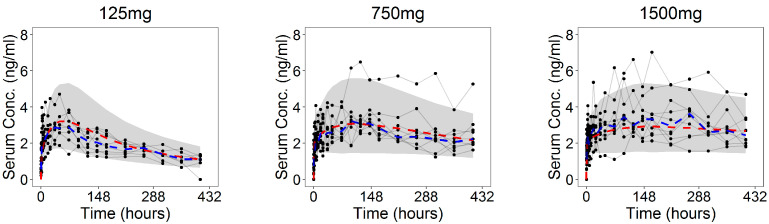
VPC plots (500 individuals for dose level) for the progesterone serum concentration profiles stratified on the dose level: The 90% confidence interval (grey area) is shown together with the observed data (black dots); dashed lines represent the theoretical (red) and experimental (blue) median.

**Figure A6:**
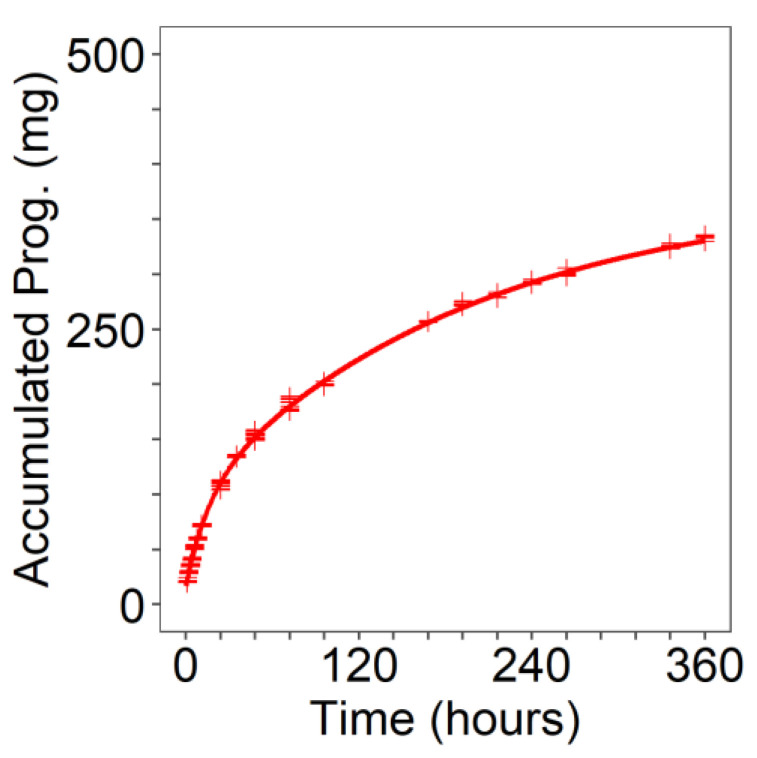
Observed in vitro individual data and fitted cumulative release profiles for test batch (batch E) at 375 mg.

**Figure A7:**
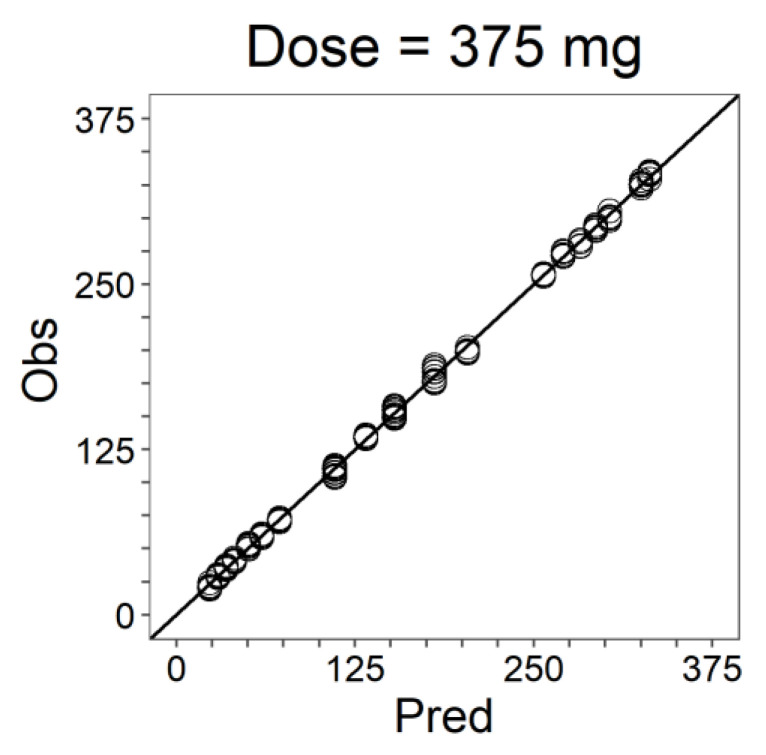
Goodness-of-fit plots for the in vitro release model for test batch (batch E) at 375 mg.

## Data Availability

Data are partially reported in the paper (aggregate information). Data not reported are part of ITF Research know-how and Intellectual property and their disclosure is restricted.

## Appendix A

**Table A1:** Parameter estimates of the population IVIVC P-ring model on dose 125, 750, and 1500 mg.

Population Parameters		
Parameter [Unit]	Typical Value (CV%)	Inter-Individual Variance (CV%)
$c_{0}$	2.4846	-
$c_{1}$	0.0212	-
$c_{2}$	$2\times 10^{-5}$	-
γ [-]	$c_{0}+c_{1}DOSE+c_{2}DOSE^{2}$	0.0592 (19)
$K_{abs,P_{0}}$ [1/h]	0.8370 (25)	0.2870 (28)
$K_{abs}$ [1/h]	0.4430 (5)	*-*
$K_{el}$ [1/h]	0.0221 (10)	0.014 (84)
*V*/*F* [L]	3880 (11)	0.0561 (28)
$K_{12}$ [1/h]	0.0195 (62)	0.2480 (95)
$K_{21}$ [1/h]	0.0703 (>100)	6.7000 (66)
**Residual variability (variance)**		
$var_{conc}$	0.2140 (3)	
$var_{amount}$	$4\times 10^{-5}$ (3)	

**Table A2:** Absolute %PEs on AUC[0-t] for the IVIVC P-ring model identified on dose 125, 750, and 1500 mg.

		Absolute PE [%]											
	Time [h]	24	48	72	96	120	144	168	216	264	312	360	408
Dose [mg]													
**125**		5.05	4.09	3.38	2.79	2.57	2.73	2.53	2.56	2.71	2.06	1.55	1.05
**750**		5.91	6.15	4.33	3.76	2.77	3.22	3.42	2.60	1.99	1.75	1.59	1.44
**1500**		3.74	2.39	5.36	5.77	3.74	2.48	2.70	2.69	2.33	1.91	1.68	1.38
**Average**		4.90	4.21	4.36	4.11	3.03	2.81	2.88	2.62	2.34	1.91	1.61	1.29

**Table A3:** Absolute %PEs on *C_max_* for IVIVC P-ring model identified on dose 125, 750, and 1500 mg.

Dose [mg]	Absolute %PE
**125**	15.00
**750**	18.60
**1500**	17.52
**Average**	17.03

## Appendix B

**Table A4:** Parameter estimates of the in vitro release model for test batch (batch E) at 375 mg.

Parameters	375 mg
	Estimate [CV%]
$P_{0}$ [mg]	17 [3]
A [mg]	77.6 [3]
B [mg]	284.4 [-]
α [1/h]	0.0648 [3]
β [1/h]	0.0051 [1]
Error(sd)	2.83 [6]
